# Strain identification and quorum sensing inhibition characterization of marine-derived *Rhizobium* sp. NAO1

**DOI:** 10.1098/rsos.170025

**Published:** 2017-03-22

**Authors:** Hong Chang, Jin Zhou, Xiaoshan Zhu, Shenchen Yu, Lu Chen, Hui Jin, Zhonghua Cai

**Affiliations:** 1 School of Life Science, Tsinghua University, Beijing 100081, People's Republic of China; 2 Shenzhen Public Platform for Screening and Application of Marine Microbial Resources, Shenzhen, Guangdong Province 518055, People's Republic of China

**Keywords:** quorum sensing inhibition, open ocean, biofilm, virulence factors, *Rhizobium* sp*.* NAO1

## Abstract

A novel strategy for combating pathogens is through the ongoing development and use of anti-quorum sensing (QS) treatments such as therapeutic bacteria or their anti-QS substances. Relatively little is known about the bacteria that inhabit the open ocean and of their potential anti-pathogenic attributes; thus, in an initiative to identify these types of therapeutic bacteria, planktonic microbes from the North Atlantic Ocean were collected, isolated, cultured and screened for anti-QS activity. Screening analysis identified one such strain, *Rhizobium* sp. NAO1. Extracts of *Rhizobium* sp. NAO1 were identified via ultra-performance liquid chromatography (UPLC) analysis. They were shown to contain *N*-acyl homoserine lactone (AHL)-based QS analogues (in particular, the *N*-butyryl homoserine lactone (C4-AHL) analogue) and could disrupt biofilm formation by *Pseudomonas aeruginosa* PAO1. QS inhibition was confirmed using confocal scanning laser microscopy and growth curves, and it was shown to occur in a dose-dependent manner without affecting bacterial growth. Secondary metabolites of *Rhizobium* sp. NAO1 inhibited PAO1 pathogenicity by downregulating AHL-mediated virulence factors such as elastase activity and siderophore production. Furthermore, as a result of biofilm structure damage, the secondary metabolite products of *Rhizobium* sp. NAO1 significantly increased the sensitivity of PAO1 to aminoglycoside antibiotics. Our results demonstrated that *Rhizobium* sp. strain NAO1 has the ability to disrupt *P. aeruginosa* PAO1 biofilm architecture, in addition to attenuating *P. aeruginosa* PAO1 virulence factor production and pathogenicity. Therefore, the newly identified ocean-derived *Rhizobium* sp. NAO1 has the potential to serve as a QS inhibitor and may be a new microbial resource for drug development.

## 1. Introduction

Biofilms are microbial communities contained within a matrix of extracellular polymeric substances (EPS) [1]. In addition to mediating a strong attachment between the cells within the biofilm and adjacent surfaces, the EPS protect the contained microbes from environmental pressures. Importantly, these biofilms can enhance the resistance of the contained microbes to antibiotics [2]. In recent decades, pathogens have developed a significant resistance to antibiotics, creating a critical need for alternative antimicrobial targets and novel therapeutic methods. One such alternative antimicrobial target is quorum sensing (QS), which is a signalling mechanism that allows communication between bacteria and that can regulate the density, toxin production and motility of a microbial population using chemical signalling molecules such as auto-inducers [3–6].

In a number of opportunistic pathogens, such as *Pseudomonas aeruginosa*, QS enhances virulence and contributes to protection against antibiotics and the host immune responses. For example, more than 6% of the genes in the *P. aeruginosa* genome are regulated by QS and are involved in the control of pathogenesis [7,8]. During the initial phase of infection, there is a low expression of virulence genes, which prevents triggering of the host immune response. Once the bacterial population reaches a sufficiently high density, QS mediates the switch from low to high virulence gene expression while concurrently suppressing the host response [9]. Therefore, a number of studies have focused on inhibiting microbial pathogenesis by disrupting bacterial QS. This strategy is neither bactericidal (it does not kill bacteria) nor bacteriostatic (it does not inhibit bacterial growth). It appears to be a particularly attractive alternative to other treatments because it does not impose a strong selective pressure, and thus bacterial resistance is less likely to develop. For this reason, the identification of compounds that interfere with the QS system is of considerable interest in an effort to develop treatments against biofilm-associated pathogens [10]. Based on this, a strategy known as QS inhibition has been developed, for which an efficient screening for QS inhibitor (QSI) agents is required.

A number of QSI bacteria and compounds have been isolated from terrestrial and aquatic environments and have been shown to have anti-QS properties that can attenuate the expression of virulence factors produced by the bacterial strain *P. aeruginosa* PAO1 [11–13]. A few marine-derived microbes, including *Halobacillus salinus* C42 [14], *Bacillus horikoshii* [15] and *B. pumilus* S8-07 [16], discovered, respectively, in sea grass, the coral *Acropora digitifera* and sediments from the Palk Bay, have been noted to have QSI potential. Interestingly, the ocean contains a broad range of microbial biodiversity in which potent bioactive compounds are produced by a number of marine organisms, indicating that the ocean can serve as an important resource in the search for novel QSI substances [17,18].

Therefore, the aim of the present study was to discover QSI compounds produced by microbes isolated from the North Atlantic Ocean. To test these compounds, *P. aeruginosa* PAO1, a representative Gram-negative pathogen that thrives in diverse terrestrial and aquatic environments and that naturally generates biofilms and produces a number of QS-regulated virulence factors (exoproteases, siderophores, exotoxins and lipases), was used as a target [7,19–21]. We screened bacteria isolated from the ocean, identified a non-toxic and potent QSI and further investigated the effects of this QSI on PAO1 biofilm formation and virulence factor expression.

## 2. Material and methods

### 2.1. Bacterial strains and culture conditions

Characteristics of the indicator strain *Chromobacterium violaceum* ATCC 12472 (ATCC®. 12472™) and *P. aeruginosa* PAO1 are listed in table 1. Both *C. violaceum* and *P. aeruginosa* were cultured in Luria-Bertani (LB) medium containing 1% peptone, 0.5% yeast extract and 0.5% NaCl, either in broth or solidified using 1% agar as necessary.

**Table 1.** List of strains used.

strain	description	purpose	source or reference
*Pseudomonas aeruginosa* PAO1	wild-type prototroph	positive control for QSI	[12]
*Chromobacterium violaceum* ATCC 12472	type strain	QSI indicator strain	[22]
*Rhizobium* sp. NAO1	potential QSI screened in this study	effect on biofilm and virulence factors	[23]
environmental bacterial isolates	The 26th Ocean Survey	QSI screening	this work

Environmental microorganism samples were collected during a cruise (from 8 June 2012 to 1 July 2012). The site of collection, known as station 26II-NAR, was located from 3°57.058′ W, 36°8.532′ N to 35°50.213′ W, 8°28.338′ N, near the equator above the mid-Atlantic ridge. The samples were collected from 30 sites at a depth of 0.5–1.0 m along this route. At each site, 1 l of water was filtered through a membrane of 47 mm diameter and 0.22 µm pore size, and the particulates collected from the filter were maintained in Zobell marine 2216 medium with 15% (v/v) glycerol. Samples were then stored at −80°C prior to use.

### 2.2. Screening for quorum sensing inhibitors

To screen for marine bacterial strains that inhibit QS-mediated violacein production, microorganisms were first collected from each filter membrane. They were then mixed with sterile saline water, vortexed and plated in serial dilutions of up to a 100-fold on 2216E agar medium (Dingguo, Shanghai, China). Plates were incubated at 30°C for 48–72 h until colonies were visible, and single bacterial colonies were isolated based on distinct colony morphologies. After incubating at 30°C for 24 h, each colony was overlaid on sterile filter paper with *C. violaceum* ATCC 12472 to observe colour development [22]. The formation of a halo without the indicator pigment was considered a positive result indicating a QSI.

### 2.3. Identification of bacterial strains

The potential QSI strains were grown overnight in 2216E broth at 30°C, and then 200 µl from each culture was transferred into a clean 1.5 ml microfuge tube and centrifuged at 6700*g* for 1 min. The flow-through in the tube was discarded, 100 µl of TE buffer was added, and the sample was mixed gently and then boiled for 10 min. The resulting supernatant contained the DNA crude extract (OD_260_/OD_230_ was more than 1.7, and OD_260_/OD_280_ between 1.8 and 2.0). The 16*S* rRNA gene, which is approximately 1500 bp, was amplified by PCR using the forward primer 27F (5′-AGAGTTTGATCCTGGCTCAG-3′) and the reverse primer 1492R (5′-GGTTACCTTGTTACGACTT-3′) [24], and sequenced at BGI-Shenzhen (BGI China, Mainland). The sequences obtained were assembled, analysed and manually edited using a CAP3 software package. The resulting sequences were compared against those from the NCBI database (http://www.ncbi.nlm.nih.gov) using BLAST analysis.

### 2.4. Extraction and identification of quorum sensing inhibition components

The potential QSI strains were incubated for 48 h in 2216E broth at 30°C with shaking at 200 r.p.m. The samples were centrifuged at 5400*g* at 4°C for 20 min, and the resulting supernatant was collected and extracted using an equal volume of ethyl acetate, with vigorous shaking for 15–20 min. The extraction was repeated twice, and the combined extracts were evaporated in a rotary evaporator at 45°C. The aqueous extract was obtained by ethyl acetate extraction [12], while the organic extract that had been dissolved in methanol was concentrated using nitrogen flow and a rotary evaporator. All the resulting extracts were sterilized using 0.22-µm filters before use.

A chromatogram of the final product was obtained using ultra-performance liquid chromatography (UPLC; Waters, USA) at 210 nm with a reverse-phase C18 column (50 × 2.1 mm). The sample was eluted with a 51 : 49 ratio of water–acetonitrile at a flow rate of 0.3 ml min^−1^. Biofilm experiments and measurements of virulence factors were conducted using a range of concentrations of the sterilized extracts.

### 2.5. Assessment of biofilm formation

Biofilm formation by *P. aeruginosa* PAO1 was quantitatively assessed as previously reported [25]. Briefly, 100 µl of bacterial culture diluted in M63 medium [26] to an initial optical density at 600 nm (OD_600_) of 0.02 was transferred into each well of a 96-well polystyrene plate (Costar 3599; Corning Life Sciences, Corning, NY, USA). To check for inhibition of biofilm formation, QSI supernatants at dilutions of 1%, 3% and 5% v/v were added to wells containing the *P. aeruginosa* cultures. To identify any bioactive compounds, 0.5% organic or 5% aqueous extracts obtained as described above were added to diluted PAO1 cultures. An untreated *P. aeruginosa* PAO1 culture was used as a negative control, and a *P. aeruginosa* PAO1 culture mixed with 10 µM of a known QSI, furanone, was used as a positive control. Cells were incubated at 37°C for 48 h without shaking. Total biofilm formation was measured using crystal violet staining [27], and the optical density was read at 595 nm using a Varioskan Flash spectral scanning multimode reader (Thermo Scientific, Pittsburgh, PA, USA). Three repeated trials to evaluate the biofilm inhibitor assay were performed, and in each experiment the data point was the average of at least 12 replicate wells.

### 2.6. Confocal laser scanning microscopy of antibiotic sensitivity of microbes in static biofilms

Confocal laser scanning microscopy (CLSM) was used to measure the tolerance of *P. aeruginosa* PAO1 in biofilms to antibiotics following treatment with QSI extracts. Each test was repeated three times, and the mean was taken to eliminate any discrepancies between measurements. *Pseudomonas aeruginosa* PAO1 was diluted into 10 ml of M63 medium supplemented with 5% of the previously recovered QSI supernatant. The mixtures were transferred to 90 mm Petri dishes, each containing a single sterilized coverslip, and then incubated at 37°C for 3 days without shaking. *Pseudomonas aeruginosa* PAO1 cultured without QSI supernatant was used as negative control. The tolerance of microbes in biofilms to antibiotics was assessed by adding 100 µg ml^−1^ kanamycin to 3-day-old biofilms. After incubating for 24 h, the biofilms were observed using CLSM after standard staining. To measure bacterial viability, a LIVE/DEAD *Bac*Light Bacterial Viability staining kit (Invitrogen) was used, as previously reported [28]. Briefly, the stock stain solution of 1.5 µl of SYTO9 and 1.5 µl of propidium iodide (PI) was diluted in 1 ml of dimethylsulfoxide, and staining was performed in the dark for 20 min at room temperature (approx. 25–27°C).

COMSTAT, a novel computer program that incorporates 10 parameters for quantitative characterization of three-dimensional biofilm images, was used to assess the biofilms. COMSTAT was written as a script in MATLAB 5.1 and was equipped with Image Processing Toolbox. Of the available parameters, we selected the three parameters of total biomass, average thickness and maximum thickness to evaluate the biofilms [29]. All images used for calculations were generated as part of the CLSM examination. Three-dimensional transmission-fluorescence images of the *P. aeruginosa* PAO1 biofilms treated with QSI extracts or kanamycin were generated using FV10-ASW2.0 Viewer (Olympus, Japan). The optical sections were 5 µm apart on the *Z*-axis and taken at 640 × 640 pixels with a 12-bit intensity resolution.

### 2.7. Measuring virulence factors

To measure the effects of the potential QSI extracts on common virulence factors of *P. aeruginosa* PAO1, assays for elastase activity and siderophore production were performed after adding 1%, 3% or 5% (v/v) aqueous extract or 0.5% organic extract at the start of the incubation [30,31]. The addition of 10 µM furanone was used as a positive control. Absorbance was measured at 495 nm and 630 nm.

### 2.8. Growth rate

To investigate the effects of the QSI extracts on the growth of *P. aeruginosa* PAO1, 5% QSI supernatant was added to the PAO1 culture. The culture was then incubated in 300 ml shaking flasks containing 100 ml LB broth with an initial OD_600_ of 0.02. Growth curves in the presence and absence of QSI supernatant were obtained by measuring the OD_600_ at the indicated time points. In addition, the count of the bacterial cells (colony-forming units, CFU) was also analysed by flow cytometry (BD FACS Calibur) according to the method of Saint-Ruf *et al.* [32]. Data acquisition and analysis were performed with a BD Accuri C6 and FlowJo software (FLOWJO LLC, Ashland, OR, USA).

### 2.9. Statistical analysis

Using SPSS software (Chicago, IL, USA), statistical significance was assessed by one-way ANOVA, where a *p*-value of 0.05 was considered significant. Each experiment was performed independently at least twice with each sample in triplicate, according to the method of Adonizio *et al.* [11].

## 3. Results

### 3.1. Isolation of anti-quorum sensing marine bacteria

**Figure 1.**
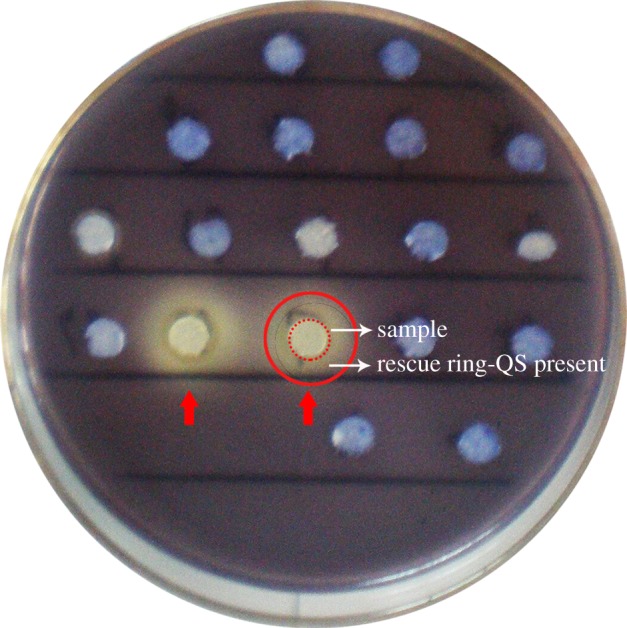
Screening of quorum sensing inhibitors. The red circular dotted line is the filter paper for sample detection, the red circular solid line is the area for QSI judgement. The formation of a transparent halo (rescue ring) was considered to indicate a potential QS inhibitor and the absence of a halo was considered negative. The red arrows refer to the positive QSI strains.

Bacteria with anti-QS properties were screened using *C. violaceum* ATCC 12472 as an indicator strain as it produces a purple pigment violacein unless its QS system is interrupted. In this system, a lack of pigmentation from the indicator organism in the vicinity of the test organism indicates a positive QSI result [22]. Hundreds of culturable bacteria were isolated from the surface waters of the North Atlantic Ocean and were screened for anti-QS activity. Ten isolates were specifically screened for colour reduction in *C. violaceum*. The isolate strain NAO1 caused the most significant reduction, in which the purple pigment of *C. violaceum* ATCC 12472 was completely eliminated (figure 1). BLAST analysis of the 16*S* rRNA gene sequence of strain NAO1 revealed a 100% sequence similarity to *Rhizobium* sp. 6001 (GenBank number JX566662.1). Therefore, this organism has been tentatively named *Rhizobium* sp. NAO1. The strain has been deposited at the China General Microbiological Culture Collection Center (CGMCC, No.12205, Beijing, China).

### 3.2. Inhibition of biofilm formation

**Figure 2.**
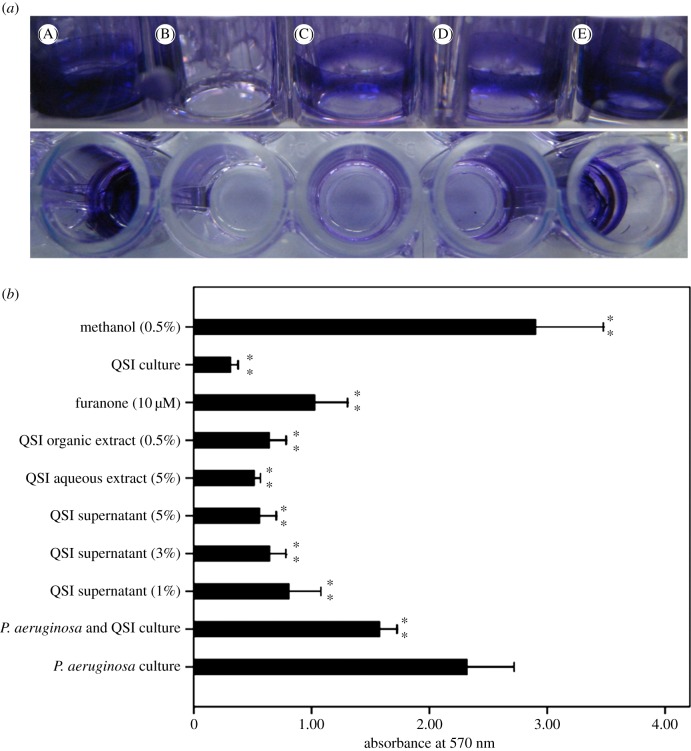
Biofilm inhibition assay. (*a*) A, *P. aeruginosa* biofilm (positive control); B, M63 broth (negative control); C, D and E, biofilm treated with 5% supernatant, 5% aqueous extract and 0.5% organic extract of QSI, respectively. (*b*) Biofilm production of *P. aeruginosa* cell culture (50 µl) in the presence of QSI supernatant (1%, 3%, 5%), aqueous extract (5%), organic extract (0.5%), furanone (10 µM), QSI culture (50 µl) and methanol (0.5%). Asterisks indicate a statistically significant difference (**p < 0.01) between experimental groups and control (*P. aeruginosa* culture).

**Figure 3.**
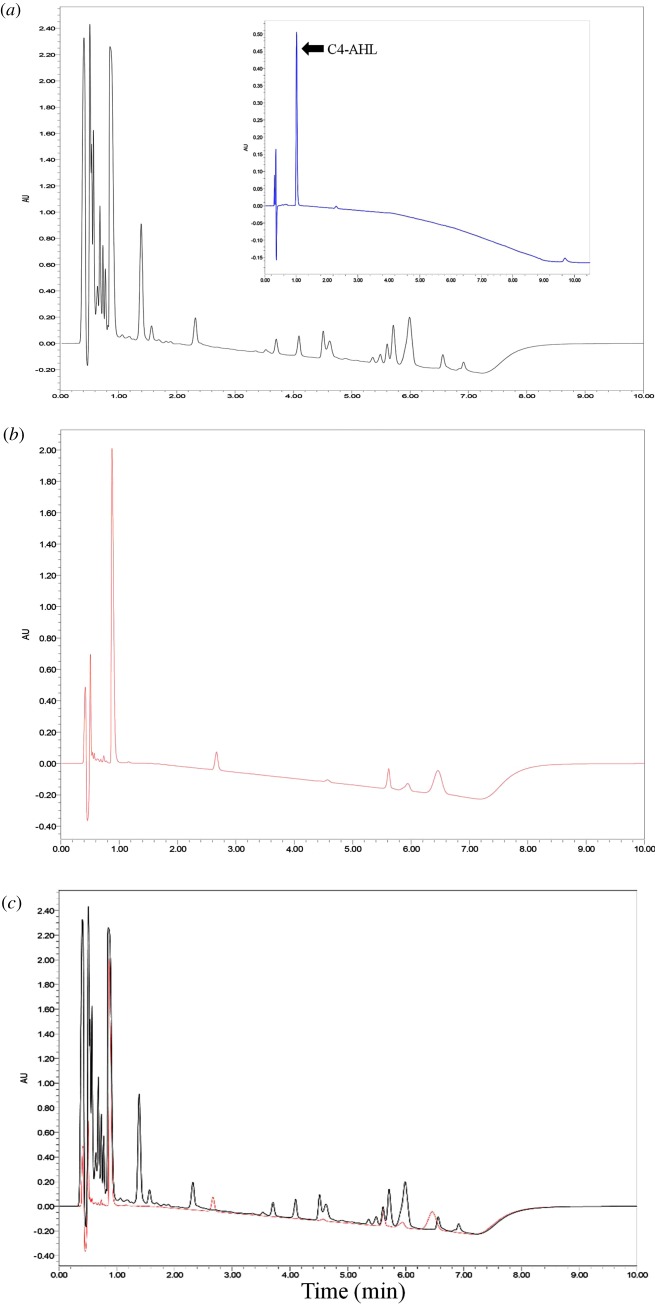
Ultra-performance liquid chromatography (UPLC) analysis of extract. (*a*) The chromatogram shows the *P. aeruginosa* PAO1 extracts, the insert picture is the standard of C4-AHL (*N*-butyryl homoserine lactone); (*b*) *Rhizobium* sp. NAO1 extracts; and (*c*) the merged picture. The black line is PAO1 and the red line is NAO1. Peaks are a function of intensity measured in milli-absorption units over time in minutes.

*Pseudomonas aeruginosa* PAO1 can form biofilms, which is a partially QS-dependent process. Therefore, the ability of extracts from isolate strain *Rhizobium* sp. NAO1 to inhibit biofilm formation by *P. aeruginosa* PAO1 was investigated. Figure 2*b* presents quantitative analysis of PAO1 biofilm inhibition. We found that addition of QSI cell culture (50 µl), QSI supernatant (1%, 3% and 5%), aqueous extract (5%) or organic extract (0.5%) to PAO1 reduced biofilm formation by 32.0% (QSI), 65.1%, 72.3% and 75.9% (three QSI supernatants), 77.9% (aqueous extract) and 72.3% (organic extract). Inhibition by different percentages of QSI supernatant occurred in a dose-dependent manner. The highest reduction in the amount of biofilm was 77.9%, which was caused by the 5% aqueous extract. Visualization by light microscopy also revealed a considerable reduction in biofilm intensity (figure 2*a*). The chromatogram results showed that the supernatant from strain *Rhizobium* sp. NAO1 likely contained analogues of the *N*-butyryl homoserine lactone (C4-AHL) compounds of *P. aeruginosa* PAO1 (figure 3). Overall, the results indicated that *N*-acyl homoserine lactone (AHL) analogues secreted by *Rhizobium* sp. NAO1 were partially responsible for the inhibition of biofilm formation.

### 3.3. Effect on biofilm stress tolerance

When targeting biofilm-forming pathogens, a number of conventional antibiotics abrogate symptoms of infection by killing free-floating bacteria that have detached from the biofilm population, but these antibiotics are ineffective against bacteria within the biofilms [33]. QS has been shown to increase resistance to antimicrobials [34]; therefore, we determined whether QSI extracts could affect multicellular biofilm defences.

**Figure 4.**
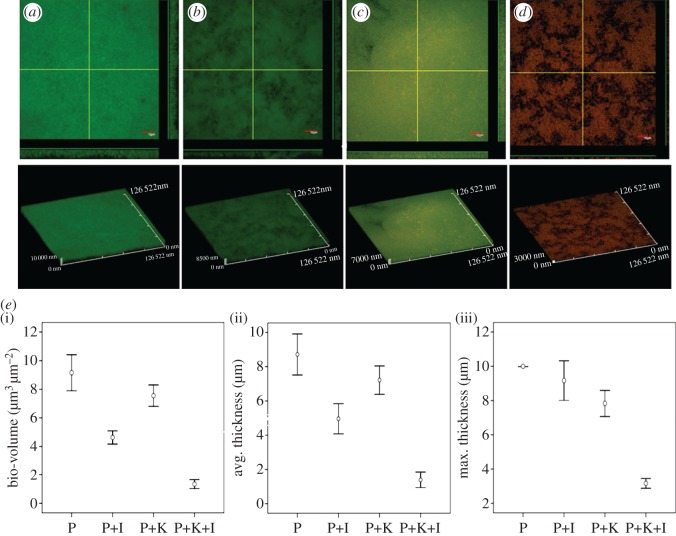
Sensitivity of QSI-treated *P. aeruginosa* biofilms to kanamycin. Confocal scanning laser microscopy (CLSM) photomicrographs of PAO1 biofilms grown in the presence or absence of QSI supernatant (5%). Three days later, the biofilm was exposed to 100 µg ml^−1^ kanamycin for 24 h. (*a*) No QSI supernatant or kanamycin; (*b*) QSI supernatant (5%); (*c*) 100 µg ml^−1^ kanamycin; (*d*) QSI supernatant (5%) plus 100 µg ml^−1^ kanamycin. Live cells were stained green and dead cells were stained red using a LIVE/DEAD *Bac*Light Bacterial Viability Kit. (*e*) COMSTAT quantification of (i) bio-volume, (ii) average thickness, and (iii) maximum thickness show that PAO1 forms thinner biofilms after being treated with kanamycin and QSI supernatant extract.

Wild-type *P. aeruginosa* PAO1 forms characteristic mature biofilms (figure 4*a*). In the presence of the 5% QSI supernatant, these biofilms became more dispersed and unstable (figure 4*b*). The susceptibility of biofilms to kanamycin was significantly enhanced following treatment with 5% QSI supernatant, resulting in degeneration of the biofilms and yielding single cells (figure 4*c*,*d*). When supplemented with QSI extracts, antibiotics efficiently penetrated and killed the cells of the biofilm to the point where only a few cells had spread.

COMSTAT analysis enabled precise evaluation of the biofilm structures (figure 4*e*). A degree of decline in the total biofilm biomass and average and maximum thicknesses was observed in both the experimental and control groups. Specifically, *P. aeruginosa* biofilms that survived treatment with a combination of QSI extracts and kanamycin showed a significant reduction in all three parameters (figure 4*e*).

### 3.4. Inhibition of *Pseudomonas aeruginosa* PAO1 virulence factors

**Figure 5.**
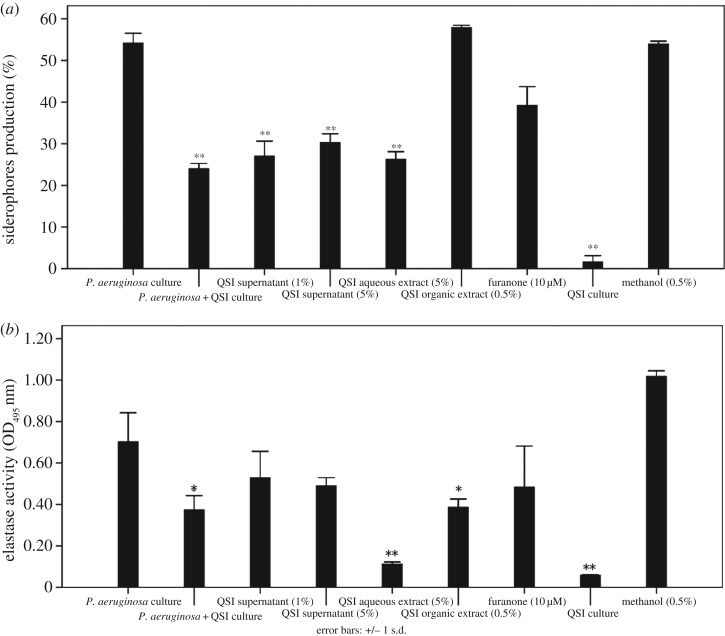
Measurement of virulence factors. Measurement of virulence factors of *P. aeruginosa* in the presence of QSI supernatant (1%, 5%), aqueous extract (5%), organic extract (0.5%), furanone (10 µM), methanol (0.5%) and QSI culture. (*a*) Siderophore production and (*b*) elastase activity. Asterisks indicate a statistically significant difference (*p < 0.05, **p < 0.01) between experimental groups and control (*P. aeruginosa* culture).

To further explore the anti-QS activities of the QSI *Rhizobium* sp. NAO1 strain, experiments were conducted measuring *P. aeruginosa* PAO1 elastase activity and siderophore production following different treatments. All tested extracts resulted in elastase activity inhibition. Specifically, PAO1 grown in 1% and 5% QSI supernatant displayed a decrease in the secretion of elastase of 65.1% and 75.9%, respectively, while 5% aqueous and 0.5% organic extracts decreased elastase activity by 77.9% and 72.3%, respectively. However, the 10 µM furanone positive control caused only a 31% reduction in elastase activity (figure 5*b*). The 1% and 5% QSI supernatant and 5% aqueous extract significantly inhibited the production of siderophores by 50.0%, 44.1% and 51.5%, respectively. The maximum reduction of 55.7% was observed in the presence of 50 µl of the QSI-strain NAO1 bacterial suspension (figure 5*a*).

### 3.5. Growth curve analysis

**Figure 6.**
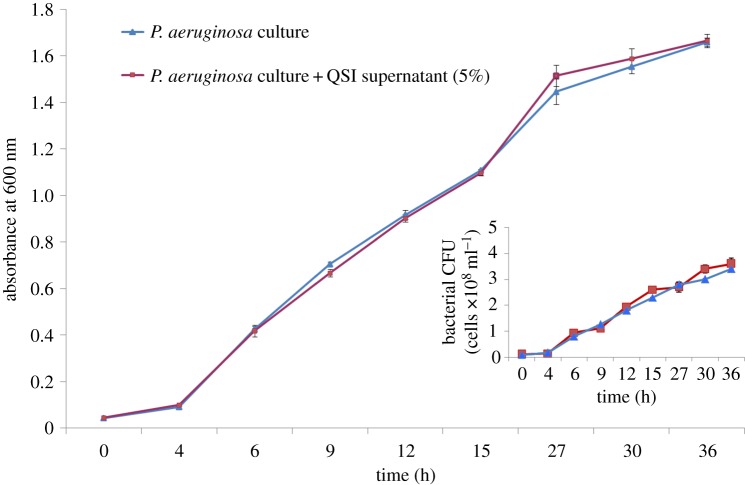
Effect of QSI on *P. aeruginosa* growth. Bacteria were grown in LB media with (red line) and without (blue line) QSI supernatant (5%). The flow cytometry results show the count of the bacterial cells (inset picture).

The effect of the QSI supernatant on *P. aeruginosa* growth is shown in figure 6. The growth curves for untreated and treated *P. aeruginosa* PAO1 were almost identical, indicating that the addition of QSI supernatant did not affect growth of *P. aeruginosa* PAO1.

## 4. Discussion

The majority of studies published on the production of QSIs by marine bacteria have focused on bacteria that were recovered from surfaces, biofilms and sediments [14,35,36]. However, a study was recently published hypothesizing that QS inhibition may be a frequently occurring characteristic in planktonic culturable marine bacteria such as *Bacillus* sp. [15,16,37] and *Vibrio* sp. [38]. These examples indicate that ocean-derived free-living bacteria may be potential sources of QSIs. In this work, the strain *Rhizobium* sp. NAO1 was collected from open water and found to have significant QSI activity, supporting the hypothesis of Thenmozhi *et al*. [15].

Accumulating evidence demonstrates that AHL-dependent QS is critical for the development of biofilms, indicating that QSIs can inhibit biofilm formation. Research by Nithya *et al*. [16] suggests that *P. aeruginosa* PAO1 biofilm maturation can be inhibited by the *B. pumilus* strain S8-07, a marine-derived bacterial species. Similar published work examining the effects of crude water extracts from *Callistemon viminalis* (1 mg ml^−1^) found that it reduced biofilm formation by as much as 89%, which has been confirmed by Adonizio *et al*. [11]. In this study, we isolated the strain *Rhizobium* sp. NAO1 and determined that it can profoundly reduce *P. aeruginosa* PAO1 biofilm production by up to 78% as shown by CLSM analyses (figure 4*e*). These finding are in accordance with previous reports. Notably, we found that the addition of either 5% aqueous or 0.5% organic extract resulted in a 77.9% and 72.3% reduction in the *P. aeruginosa* PAO1 biofilm, respectively, indicating that active anti-QS compounds are present in both aqueous and organic extracts (figure 2). Based on this observation, it is plausible that the active QSI source may contain both enzymatic and non-enzymatic compounds such as analogues of QS molecules [39]. In terms of enzymatic degradation, similar results have been reported regarding homologues of the AiiA enzyme, which is used by microorganisms such as *Agrobacterium tumefaciens*, *Klebsiella pneumoniae* and *Rhodococcus* sp. [2,40]. In terms of non-enzymatic compounds, an active QSI substance was determined to be an AHL-based analogue that was in competition with the bacterial AHL system. In this work, we determined that *Rhizobium* sp. NAO1 has the potential to produce C4-AHL analogues (figure 3), by which it inhibits biofilm formation by *P. aeruginosa* PAO1. Over the past decade, AHL-based analogues have been extensively developed as QS modulators or anti-biofilm agents [41]. Consistent with our findings, Teasdale *et al*. [14] found that the QSI properties exhibited by the marine bacterium *H. salinus* C42 were present in the solvent phase, in which the solvent was ethyl acetate. The biological activity was associated with QS analogues that act as QS-antagonists by competing with AHL for receptor binding. In this study, inhibition of the QS system by bacterial extracts was observed, although the composition of the compounds responsible for this phenotype is complex. There are two possible explanations: (i) multiple chemicals produced by the bacteria cause unique effects at a variety of points in the QS system and/or (ii) these compounds are not directly acting on the AI-1 system but, instead, are interfering with a global regulator of QS such as AI-2 [42]. However, further studies aimed at purifying and characterizing *Rhizobium* sp. NAO1 extracts are necessary in order to elucidate the mechanisms of action responsible for the inhibitory properties.

In addition to anti-biofilm activity, *Rhizobium* sp. NAO1 also inhibited the production of *P. aeruginosa* PAO1 virulence factors such as siderophores and elastase (figure 5). These results are similar to findings by Adonizio *et al*. [11], in which six south Florida medicinal plants were examined for anti-PAO1 properties and found to be anti-QS. A report published by Park *et al.* [43] showed that the *Streptomyces* strain M664 produces an AHL-degrading acylase enzyme that degrades AHL-regulated elastase and total and *LasA* proteases by 43%, 60% and 50%, respectively. In addition, Musthafa *et al.* [44] demonstrated that the marine-derived *Bacillus* sp. SS4 inhibited AHL-regulated production of *P. aeruginosa* PAO1 virulence factors. Overall, these studies strongly suggest that certain QSI bacteria, or active compounds isolated from these bacteria, have the potential to lessen the virulence of *P. aeruginosa* by interfering with the proper production of virulence factors. This may also be applicable to other AHL-producing Gram-negative bacteria. The prevailing hypothesized mechanism of action for the way in which QS increases pathogen virulence suggests that virulence factor production is inhibited, thus enabling the bacteria to remain undetected by the host immune system at a low cell density, since bacterial virulence factors are often immunogenic. However, once the bacterial population reaches a certain density, such as those observed in biofilms, QS triggers the expression and secretion of virulence and host-damaging factors [45]. Therefore, we hypothesized that the inhibition of elastase activity and siderophore production by *Rhizobium* sp. NAO1 occurs via interference with QS activity because these virulence factors are under the control of the *lasI-lasR* and *rhlI-rhlR* systems [11].

An important phenomenon observed in this study was that the QSI extracts acted in a synergistic manner with kanamycin treatment; *P. aeruginosa* biofilms treated with 5% QSI supernatant were thinner and more dispersed relative to the positive control, and the combined use of kanamycin and QSI extracts resulted in the killing of the majority of the bacteria. *Pseudomonas aeruginosa* PAO1 biofilms treated with QSI extracts were significantly thinner than untreated biofilms, suggesting that this may be a possible approach for increasing the susceptibility of *P. aeruginosa* PAO1 to antibiotic treatment. In agreement with our results, You *et al*. [46] isolated *Streptomyces albus* from marine sediments collected from the South China Sea and found that this bacterium significantly increased *Vibrio harveyi* sensitivity to antibiotics and interfered with biofilm activity*.* Another report by Bakkiyaraj *et al*. [47] described inhibition of *P. aeruginosa* PAO1 biofilm formation by coral-associated isolates. Hence, the use of QSI extracts in conjunction with antibiotics may enhance pathogen killing through two possible mechanisms: (i) the combination of a QSI and antibiotic could disrupt the multicellular structure of the biofilm, thus rendering the bacteria susceptible to eradication by antibiotics and/or (ii) biofilm architecture is primarily dependent on the secretion of an AHL-regulated exopolymeric substance [48]. Therefore, inhibiting AHL can prevent the synthesis of these exopolymeric substances, leading to a loose *P. aeruginosa* PAO1 biofilm structure [6]. In this study, QSI extracts reduced biofilm formation and the production of virulence factors, specifically siderophores and elastase, and rendered *P. aeruginosa* biofilms more susceptible to kanamycin. Thus, QSI molecules are novel antibacterial agents that can enhance the bactericidal function of antibiotics. However, the effectiveness, stability and degradability of QSI extracts under natural conditions require further exploration.

In summary, this study uncovered the anti-QS activity of a marine bacterial species isolated from samples collected from the open ocean. Extracts of *Rhizobium* sp. NAO1 were antagonistic to *P. aeruginosa* PAO1 QS and affected QS-regulated phenotypes, including biofilm formation, virulence factor production and antibiotic susceptibility. It is possible that analogue molecules produced by *Rhizobium* sp. NAO1 competed with the auto-inducers produced by *P. aeruginosa* PAO1. Interestingly, extracts of *Rhizobium* sp. NAO1 did not affect the growth of *P. aeruginosa* PAO1. These characteristics may help develop new classes of anti-QS compounds with broad-spectrum activity, in addition to catalysing an increased exploration of novel functions of bacterial species residing in the ocean.

## Data accessibility

Data are available from the Dryad Digital Repository: http://dx.doi.org/10.5061/dryad.27630 [49].
